# Physicians' Perceptions and Practices Regarding Patient Reports of Multiple Chemical Sensitivity

**DOI:** 10.5402/2011/838930

**Published:** 2011-09-07

**Authors:** Pamela Reed Gibson, Amanda Lindberg

**Affiliations:** Department of Psychology, James Madison University, MSC 7704, Harrisonburg, VA 22807, USA

## Abstract

Ninety physicians practicing in the state of Virginia USA completed a mail survey regarding Multiple Chemical Sensitivity (MCS). Survey questions addressed demographics; familiarity with MCS; etiology; overlapping conditions; accommodations made for patients and practices regarding evaluation, treatment, and referral. A little over half of respondents were familiar with MCS. Under a third had received any medical training regarding chemical sensitivity, only 7% were “very satisfied” with their knowledge, and 6% had a treatment protocol for the condition. Participants cited a range of etiologies and overlapping conditions including asthma, Reactive Airway Dysfunction Syndrome (RADS), Sick Building Syndrome (SBS), Chronic Fatigues Syndrome (CFS), and Fibromyalgia. Physicians infrequently considered chemicals as a cause of illness when seeing new patients. Evaluation techniques included interviews, blood work, immune profiles, and allergy testing. Interventions recommended included chemical avoidance, alterations in the home environment, diet restrictions, the use of air filters, and referrals to outside specialists.

## 1. Introduction

Persons who experience Multiple Chemical Sensitivity (MCS), also referred to as chemical intolerance, environmental illness, and chemical hypersensitivity [1], are a medically underserved group making up 12.6% of the US population [2], with 4% experiencing the symptoms daily [3]. The condition has been studied in a number of other countries as well, including Japan [4, 5], Germany [6], Sweden [7, 8], and The Netherlands [9]. And in Canada 2.4% of Canadians aged 12 and over have been diagnosed with MCS [10]. Individuals with MCS report experiencing disabling symptoms as a result of low-level exposures to chemicals in ambient air generally tolerated by a majority of the population. The need for chemical avoidance limits their access to environments where such exposures might occur, such as libraries, medical offices, grocery stores, community meetings, and places of worship [11]. Though the diagnosis of Multiple Chemical Sensitivity has been the subject of a detailed report commissioned by the Canadian Human Rights Commission [12], the condition continues to be surrounded by medical controversy and uncertainty regarding its label, causes, and indicated treatments. Unlike chronic fatigue (myalgic encephalomyelitis or ME in the UK), which now receives some recognition and study from the medical profession, MCS remains a marginalized condition in mainstream medical practice and patients report mixed experiences when requesting medical help. 

McColl et al. found that persons with disabilities in general had three times the unmet medical needs of nondisabled people and that disabled respondents with unmet needs had seen an increased number of providers, but still perceived the system to be inadequate [13]. Because physicians are often unfamiliar with and/or do not believe in MCS and because their offices may contain chemical barriers, individuals often receive inadequate medical attention. Patients report experiencing considerable iatrogenic harm due to unmet medical needs, delays in correct diagnosis, or treatment for the wrong condition [14]. Gibson et al. found that patients experimented with between 24 and 37 different treatments, thus spending considerable time and money on interventions that may or may not be helpful [15]. To date, only one researcher has studied physicians' views regarding MCS [16]. The purpose of the current study was to examine practicing physicians' current views and practices relating to multiple chemical sensitivities.

## 2. Method

After receiving approval from our university Institutional Review Board, we mailed a survey that included informed consent to a random sample of 1000 US physicians licensed and practicing in the state of Virginia. Questions included information about the participants' medical specialty, practice setting, and other demographics; any training or education received regarding chemical sensitivity; amount of experience with patients reporting sensitivities; degree of and satisfaction with knowledge regarding MCS; personal beliefs about the causes and appropriate treatments for the condition; treatment protocols; and referral practices. One reminder was sent.

## 3. Results

### 3.1. Demographics and Training

Participants included 90 physicians licensed and practicing in Virginia with a mean of 15.5 years in practice. Overall, 26 specialties were included; most commonly represented were family practice and internal medicine. Other specialties included gynecology, emergency medicine/urgent care, anesthesiology, diagnostic radiology, ophthalmology, dermatology, general surgery, podiatry, otolaryngology, occupational/environmental medicine, cardiology, pediatrics, orthopedics, physical medicine and rehabilitation, rheumatology, medical acupuncture, geriatrics, oral and maxiofacial surgery, neonatology, pulmonary medicine, urology, pathology, nephrology, and oncology. Physicians reported having seen a median of 3 patients with chemical sensitivities in the past year and 10 over the course of their careers. When asked how familiar they were with MCS, 9% responded “very unfamiliar,” 36% “somewhat unfamiliar,” 48% “somewhat familiar,” and 8% “very familiar.” Physicians reported gaining knowledge about MCS from a variety of sources, including other health providers (51%), journal articles (47%), formal education/medical school (30%), the media (16%), mentors/experts (13%), professional conferences (9%), and books (4%). Respondents rated their level of satisfaction with their current knowledge of MCS as “not at all satisfied” (35%), “somewhat satisfied” (59%), or “very satisfied” (7%).

### 3.2. Physicians Perceptions

**Figure 1:**
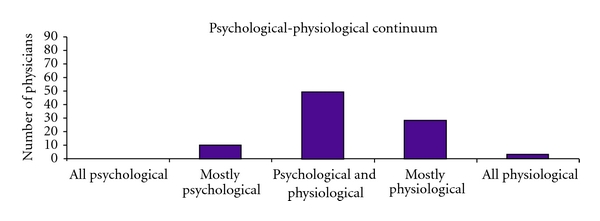
Physicians' views regarding etiology of MCS.

When asked whether they believed chemical sensitivity to be a medical or psychological condition, over half saw it as a combination, and there was a slight skew towards physiological etiology as seen in Figure 1. No respondents endorsed a purely psychological etiology. However, physicians' beliefs varied regarding the causes of MCS. When asked to select which commonly theorized causes played a role in the development of MCS, almost all respondents selected all the options given. “Multiple low level chemical exposures over time” was selected most often, endorsed by 90% of physicians. “One large chemical exposure” and “genetics” were each endorsed by approximately three quarters of respondents, as were “psychological factors,” “stress,” and “elevated risk perception.”

In those who have already developed MCS, physicians often saw gender (51%), geographic location (41%), educational level (40%), and socioeconomic status (41%) as influencing the development of the condition. Fewer respondents considered age (31%) and race (21%) to be influencing factors. 

Physicians also listed dozens of conditions that they believed overlap with MCS. The most commonly listed were asthma (91%), Reactive Airway Dysfunction Syndrome (79%), Sick Building Syndrome (71%), Chronic Fatigue Syndrome (62%), and fibromyalgia (60%). In an open-ended section, physicians listed dozens of other conditions, including allergy, rash/dermatitis, rhinitis, headaches/migraines, Lyme disease, irritable bowel syndrome, lupus, autism, and autoimmune disorders. Psychological factors were also noted, including depression, generalized anxiety, posttraumatic stress disorder, and hopelessness.

### 3.3. Physicians' Practices

**Figure 2:**
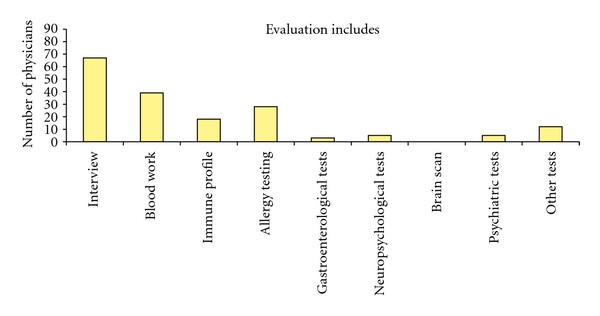
Evaluation techniques used by physicians for patients with MCS.

The majority (87%) of physicians reported only rarely or sometimes considering chemicals as the cause of a patient's illness, and only 6% reported having a defined treatment protocol for this population. The only evaluation procedure used by more than half of respondents was the patient interview. See Figure 2 for other evaluation techniques used.

After determining that a patient was experiencing sensitivity to chemicals, some physicians reported making accommodations for the patient within their office. Forty-two percent reported refraining from using fragrances or problematic personal care products when visiting with the sensitive patient, and 21% reported lessening the use of chemical cleaners within the office space. Only 15% alerted patients to chemical changes in the office environment. Few reported meeting in a safer location for the patient (12%) or making home visits (2%). 

**Figure 3:**
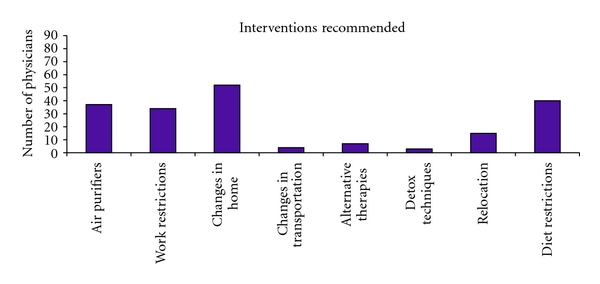
Interventions suggested by physicians for patients with MCS.

**Figure 4:**
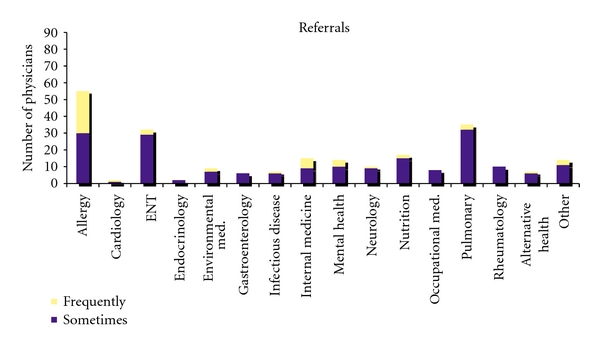
Outside referrals made by physicians for patients with MCS.

Specific interventions recommended by physicians included chemical avoidance (82%), alterations in the home environment (64%), diet restrictions (49%), or air purifiers in the home (46%). Twenty percent commonly referred patients for psychiatric evaluation or counseling. See Figure 3 for other interventions suggested. Some physicians treated patients themselves, but also made referrals to outside specialists (see Figure 4). Most commonly these referrals were to allergists, ENT specialists, and pulmonary specialists. 

## 4. Conclusions

Though 97% of respondents in this study have had patients reporting chemical sensitivities, only 6% had a treatment protocol for this condition. With prevalence of MCS at approximately 13% of the US population [2] and patients reporting accessing a mean of eight physicians each over the course of their illness [1] it is clear that there is a need for informed medical help for people with chemical sensitivities. Unfortunately, only 30% of physicians in this sample had received any training regarding MCS in medical school. And given that only 13% reported frequently considering chemicals when diagnosing health problems in new patients, missed cases of MCS may result in incorrect treatment and possible iatrogenic harm. 

People with MCS may lose employment due to sensitivities and have a need for Social Security Disability Income (SSDI). Yet, 49% of respondents indicated that they were unlikely to accept a patient applying for worker's compensation or SSDI. Similarly, 62% were unlikely to accept a patient involved in accommodation-related job litigation. 

McColl et al. report that the barriers to receiving treatment for persons with disabilities include the physical layout of the practice and its barriers, health care providers' attitudes toward disability, providers' expertise regarding their disability, and other systemic factors [13]. In addition to these barriers, persons with MCS face the chemical barriers that are common in commercial settings, including chemical cleaners, pesticides, fragrance on medical personnel, and other exposures that may require mitigation in order for persons to gain medical access. Fortunately, some respondents made accommodations for persons with MCS to visit their offices. 

Respondents made referrals to a large number of specialists, highlighting the need for education about MCS across medical specialties. Research into the development of effective treatment protocols is necessary in order for patients with MCS to receive well thought out care. Given the controversy regarding MCS etiology, it is uncertain what type of training physicians in this study received. However, it is important that health practitioners in training attend to the growing body of research on physiological mechanisms for MCS and not simply dismiss the condition as psychogenic. 

In addition, given that a high percentage of people with MCS attribute the onset of their illness to chemical exposure, there is a need for greater understanding of the toxic effects of chemicals in ambient air. It is of concern that physicians only infrequently considered chemicals as a source of illness. Henry called for nurses and physicians to attend to pesticide-induced illness over 15 years ago [17], and physicians have long called for attention to conditions caused by chemical exposure [18, 19]. Some attention to toxicology would not only include those with sensitivity syndromes in mainstream medical care, but would move toward acknowledging the contribution of toxics to common conditions such as asthma and cancer that are still not well understood [19]. 

Nursing researchers have addressed the issue of MCS [20–24], thus educating nursing personnel regarding the characteristics and needs of this population. Nurses can assist with providing accommodations, designing treatment protocols, and conducting research on this under-addressed condition. Doing so will aid in making health care provision more inclusive for persons who have been underserved and denied necessary health care and assistance in living. Accessible offices, educated health care providers, and willingness to assist with disability applications and community-based accommodations would address crucial needs and reduce suffering and obstacles in the lives of persons with environmental sensitivities. 

This study suffers from a low response rate, yet the rate may be an indication of the position of MCS in mainstream medicine, that is, it may be that responders are actually those with the most open attitudes toward the condition. For example, one nonrespondent sent us a note back with an empty survey that read, “Don't waste my time any more.” And the Virginia State Medical Association refused to allow us to hand out surveys at their annual meeting because our research was “not consistent” with the goals of their meeting. Despite this resistance, results are useful for examining how physicians who do recognize and address MCS treat the condition and to whom they make referrals. This base of responsive physicians should be increased in order to track and improve access, prevention, and medical treatments available to persons with MCS [10]. The spreading phenomenon associated with MCS dictates that patients may have a tendency to worsen in the absence of intervention. To allow a contested illness to continue to deteriorate the lives of those who experience it without making efforts to understand, prevent, and treat the condition is irresponsible. Yet MCS remains on the margins, creating a struggle for survival and access in those who experience it.
